# Uncertainty Makes Me Emotional: Uncertainty as an Elicitor and Modulator of Emotional States

**DOI:** 10.3389/fpsyg.2022.777025

**Published:** 2022-03-08

**Authors:** Jayne Morriss, Emma Tupitsa, Helen F. Dodd, Colette R. Hirsch

**Affiliations:** ^1^Centre for Integrative Neuroscience and Neurodynamics, School of Psychology and Clinical Language Sciences, University of Reading, Reading, United Kingdom; ^2^College of Medicine and Health, University of Exeter, Exeter, United Kingdom; ^3^Institute of Psychiatry, Psychology & Neuroscience, King’s College London, London, United Kingdom; ^4^South London and Maudsley NHS Foundation Trust, London, United Kingdom

**Keywords:** uncertainty, risk, ambiguity, emotion, negative, positive

## Abstract

Uncertainty and emotion are an inevitable part of everyday life and play a vital role in mental health. Yet, our understanding of how uncertainty and emotion interact is limited. Here, an online survey was conducted (*n* = 231) to examine whether uncertainty evokes and modulates a range of negative and positive emotions. The data show that uncertainty is predominantly associated with negative emotional states such as fear/anxiety. However, uncertainty was also found to modulate a variety of other negative (i.e., sadness/upset, anger/frustration, and confusion) and positive (i.e., surprise/interest and excited/enthusiastic) emotional states, depending on the valence of an anticipated outcome (i.e., negative and positive) and the sub parameter of uncertainty (i.e., risk and ambiguity). Uncertainty increased the intensity of negative emotional states and decreased the intensity of positive emotional states. These findings support prior research suggesting that uncertainty is aversive and associated with negative emotional states such as fear and anxiety. However, the findings also revealed that uncertainty is involved in eliciting and modulating a wide array of emotional phenomena beyond fear and anxiety. This study highlights an opportunity for further study of how uncertainty and emotion interactions are conceptualised generally and in relation to mental health.

## Introduction

Emotions form a vital aspect of the human condition and consequently have significant implications for health and well-being (Davidson, 1998; LeDoux, 1998). Given the complex and multifaceted nature of emotion, there is substantial variation in the literature concerning conceptual definitions of emotional phenomena (Ekman and Davidson, 1994). However, there is a general consensus that emotion comprises changes in behaviour, bodily responding (i.e., *via* the autonomic nervous system) and subjective experiences to salient internal or external events (Frijda, 1986). Prior research has defined and measured emotional phenomena categorically, encompassing specific and discrete emotional states (i.e., happiness, sadness, anger, and fear), or dimensionally, traditionally across two continuums: valence (negative-positive) and arousal (Mauss and Robinson, 2009). In recent years, empirical evidence from cross-cultural research has extended dimensional models of emotion, recommending that four major dimensions are required to support adequate discrimination of 24 emotion terms: valence, arousal, power/control, and unpredictability (Fontaine et al., 2007, 2013). Whilst progress has been made to define and understand the dimensions that support emotional phenomena (Cowen and Keltner, 2017), there is a limited literature on the role of uncertainty (an umbrella term for unpredictability, risk, ambiguity, novelty, etc, for discussion see Carleton, 2016) as a dimension that elicits and modulates emotional states (Roseman, 1984; Smith and Ellsworth, 1985; Fontaine et al., 2007).

Akin to emotion, uncertainty is a common and interconnected aspect of human experience. Uncertainty generally refers to when something is unknown, or when there is a lack of information concerning the probability of future events and their possible outcomes (i.e., awaiting the result of an academic exam or medical test) (Carleton, 2016; Morriss et al., 2019). Uncertainty can be broadly parsed out into two sub parameters: risk [also known as irreducible uncertainty, first-order uncertainty, expected uncertainty (Ellsberg, 1961; Angela and Dayan, 2005; Bach et al., 2011; Kobayashi and Hsu, 2017)] and ambiguity [also commonly referred to as second-order uncertainty or unexpected uncertainty (Ellsberg, 1961; Angela and Dayan, 2005; Soltani and Izquierdo, 2019)]. Risk occurs when there is known and reliable uncertainty related to potential outcomes. For example, when rolling a die with six sides, we know that the die will land on one of the six sides. However, there is uncertainty as to what exact side the die will land on each time we roll it. Ambiguity occurs when information is unclear or unknown making it difficult to estimate potential possibilities or outcomes. For example, when rolling a die with an unknown number of sides, we know that the die will land on a side, but we do not know the exact side it will land on or the number of sides the die could potentially land on. Importantly, information can sometimes be gathered to reduce ambiguity (i.e., revealing how many sides the die had in the example above). However, in some instances relevant information cannot be gathered to reduce ambiguity.

Both animals and humans are driven to minimise uncertainty, in order to conserve energy and accurately estimate the occurrence of motivationally relevant events (i.e., avoidance of predation, receiving comfort from conspecifics) (Hirsh et al., 2012; Peters et al., 2017). Current theoretical models posit that uncertainty is aversive in and of itself and is consequently more likely to engage the behavioural inhibition system responsible for stress and associated negative emotional states, particularly anxiety and fear (Gray, 1990; Hirsh et al., 2012; Brosschot et al., 2016; Carleton, 2016). Because of this theoretical stance, previous research has predominantly focused on uncertainty in relation to anxiety and fear, rather than other negative (i.e., frustration, anger, and sadness) or positive (i.e., surprise and excitement) emotional states (Anderson et al., 2019). Yet, earlier research suggests that appraisals of uncertainty may not only play a role in evoking anxiety and fear but also surprise, and to some extent sadness and frustration (Roseman, 1984; Smith and Ellsworth, 1985; Baas et al., 2012). Moreover, a limited literature has demonstrated that uncertainty intensifies negative emotional states (Bar-Anan et al., 2009) and dampens positive emotional states (van Dijk and Zeelenberg, 2006). While significant advancements have been made toward developing a conceptual understanding of how uncertainty and emotion intersect (Roseman, 1984; Smith and Ellsworth, 1985; Fontaine et al., 2013; Anderson et al., 2019), there remain questions as to whether uncertainty evokes and modulates a broader range of negative and positive emotions generally, depending on the anticipated valence of an outcome (positive or negative), and depending on the sub parameter of uncertainty (risk or ambiguity). It is both timely and imperative to address these nuances in how uncertainty and emotion operate, given the ubiquity of uncertainty in daily life and the role that uncertainty distress and emotion plays in a number of mental health disorders (for review see: Birrell et al. (2011), Einstein (2014), Carleton (2016), Hirsch et al. (2016), Tanovic et al. (2018), McEvoy et al. (2019), Pulcu and Browning (2019)).

In the present study, we developed an online survey to examine whether uncertainty: (1) generally elicits and modulates negative and positive emotional states, (2) elicits negative and positive emotional states differently depending on the anticipated valence of an outcome (positive or negative), and (3) elicits negative and positive emotional states differently depending on the sub parameter of uncertainty (risk or ambiguity). Based on prior literature outlining uncertainty as aversive (Hirsh et al., 2012; Grupe and Nitschke, 2013; Carleton, 2016), we hypothesised that uncertainty in general and uncertainty when anticipating a negative outcome would evoke significantly more negative, over positive, emotional states, particularly fear, and anxiety. Secondly, we hypothesised that uncertainty when anticipating a positive outcome would elicit both positive and negative emotional states to a similar degree (Anderson et al., 2019). Thirdly, we hypothesised that there may be differences in the extent to which uncertainty evokes negative and positive states depending on the sub parameter of uncertainty (i.e., risk and ambiguity). Finally, we hypothesised that experiencing uncertainty in everyday life *via* the questionnaire would be significantly associated with heightened intensity of existing negative emotional states (i.e., fear/anxiety, sadness/upset, angry/frustrated, and disgust), and reduced intensity of existing positive emotional states (i.e., happiness/joyful and excited/enthusiastic).

## Materials and Methods

### Participants

A total of 231 participants responded to the online survey (*M* age = 28.92 years, SD = 12.47; 172 Female, 47 Male, 8 Other, 4 Unknown/Not specified; Sexual Orientation: 167 Heterosexual, 43 LGBTQ+, 21 Unknown/Not specified; Ethnicity: 152 White, 25 Asian, 15 Black/African/Caribbean, 12 Latinx, 5 Middle Eastern, 4 Multi-ethnic, 1 Other, 17 Unknown/Not specified; Nationality: 105 European, 85 North American, 16 Asian, 5 South American, 3 African, 2 Australasian, and 15 Unknown/Not specified)^1^. Participants were recruited *via* poster advertisements distributed across various social media platforms, such as Twitter and Instagram, alongside several Facebook pages, including local community groups, research-related communities, and groups featuring psychology/health topics. Participants were eligible to take part in the survey provided they were 18 years or older. Sensitive information concerning past or present experience of mental health conditions (i.e., anxiety and depression) was not obtained, or controlled for, in the current sample. Participation was voluntary and all participants provided virtual informed consent prior to accessing the online survey. No incentives were offered, nor were individuals remunerated for their participation. The study procedure was approved by the University of Reading Research Ethics Committee.

### Materials

#### Uncertainty and Emotion Questionnaire

We developed a novel questionnaire to examine the interplay between uncertainty and a range of emotional experiences. The uncertainty and emotion questionnaire comprised 14 questions in total which asked participants to indicate the following: emotions they commonly associate with different parameters of uncertainty, behaviours used to manage uncertainty in daily life (not reported here), the degree to which encountering uncertainty in daily life modulates the self-reported intensity of existing emotional states, and to provide brief, written descriptions of specific situations that have previously evoked negative and positive emotions (not reported here). The questions relevant to the current study are summarised in further detail below (see the Supplementary Material for the full questionnaire).

##### Emotions Associated With Different Parameters of Uncertainty

Five questions were designed to examine the reported frequency of discrete positive and negative emotions in relation to five distinct uncertainty parameters. Participants could select one or more of the following emotion categories in response to each of the five questions: happiness/joyful, sadness/upset, fearful/anxious, disgusted, angry/frustrated, surprised/interested, excited/enthusiastic, and confused. The option “other, please specify” was provided to give participants the opportunity to disclose any other relevant emotions that were not covered by the eight existing emotion categories.

The first question asked participants to indicate the emotions they commonly associated with “*uncertainty **generally***.” The following two questions specifically focused on uncertainty in relation to the valence of the potential outcomes, with one question asking participants to indicate the emotions they commonly associated with “*uncertainty in relation to potentially **negative** outcomes (i.e., exam situations and job applications)*” and the other referring to *“uncertainty in relation to potentially **positive** outcomes (i.e., exam situations and job applications)*.” Given that the focus is on the differing valence of the potential outcome (negative versus positive), both questions made reference to the same example situations to keep the context consistent. The final two questions asked participants to select the emotions they commonly associated with uncertainty in relation to the sub-parameters of risk and ambiguity, respectively. The sub-parameter of risk was phrased as “*uncertainty when you **can predict** the possible outcomes*” with the example “*i.e., in a job application, you know that you will either be successful or unsuccessful*.” Conversely, the sub-parameter of ambiguity was phrased as “*uncertainty when you **can’t predict** the possible outcomes because there are many potential outcomes*” with the example “*i.e., your employer is considering merging departments, potentially resulting in a change of contract type, new role, promotion, or redundancy*.”

##### Uncertainty as a Modulator of Existing Emotional States

Six questions were designed to assess the modulatory impact of uncertainty on the experience of six existing emotional states in daily life. Participants were asked to indicate the degree to which encountering uncertainty would impact the intensity of an existing emotional state on a 5-point Likert scale (1 = weaker, 5 = stronger). Example item: “*If you were feeling **sad/upset** would encountering uncertainty in your day to day life make this emotional state*…”. The six discrete emotion categories were: happy/joyful, sad/upset, fearful/anxious, disgusted, angry/frustrated, and excited/enthusiastic.

For this study, the Cronbach’s Alpha for the positive questions combined (happy/joyful and excited/enthusiastic) was α = 0.73 and for the negative questions combined (sad/upset, fearful/anxious, disgusted, and angry/frustrated) was α = 0.87.

### Validity Check

All participants (*N* = 231) responded to all key questions in the survey (note that participants did not have to disclose demographic information). Approximately 92.21% (213) and 87.33% (203) of participants provided a written response to the open-ended questions (not analysed or reported here) in the uncertainty and emotion questionnaire, respectively, suggesting a good level of engagement.

### Procedure

Participants responded to an online study advertisement *via* various social media platforms (i.e., Facebook, Twitter, and Instagram) and followed a secure link that led them to the online survey hosted on JISC Online Survey^2^. Following a brief description of the study, participants who provided consent first responded to initial demographic questions, including: date of birth, gender identity, ethnicity, nationality, and sexual orientation. This was followed by the completion of 14 questions that comprised the uncertainty and emotion questionnaire. Finally, participants completed three other self-report questionnaires concerning uncertainty and mood as part of the online survey which are not included here. The questionnaires and questionnaire items followed the same order for all participants. The survey took approximately 20 min to complete.

### Analyses

Statistical tests were conducted using SPSS 27.0 (SPSS, Inc., Chicago, IL, United States). In relation to emotions commonly associated with the five parameters of uncertainty, responses to each of the nine emotion categories (including “other”) were coded either with a “1” (“yes”) if the participant indicated the experience of this particular emotion, or “0” (“no”) if they did not select this emotion category. The total frequency of each emotion was calculated by the sum of “1” (“yes”) responses across participants for each uncertainty parameter. We opted to only include the eight discrete emotion categories in the main analyses given that the “other” emotion category was not found to be used often across the five uncertainty parameters and participant responses occasionally described similar or equivalent emotions to those already provided as an option (see Supplementary Table 1 for the frequencies of “other” emotion responses across the five uncertainty parameters).

Given the binary nature of the emotion outcome variable (0 or 1), differences in the frequency of reported emotions were assessed using Cochran’s *Q* tests, a non-parametric equivalent to the repeated-measures ANOVA^3^. In the instance of a significant main effect, *post hoc* comparisons were conducted using pairwise McNemar’s tests. The Bonferroni-Holm correction (Holm, 1979), a powerful sequentially rejective multiple testing procedure that strongly controls the family wise error rate, was applied to the McNemar pairwise tests to account for multiple comparisons. Effect sizes for Cochran *Q* tests were estimated using the chance-corrected measure (“*R*”) devised by Berry et al. (2007) and interpreted using the following criteria: 0–0.2 (small), 0.2–0.5 (moderate), 0.6–0.8 (relatively large), 0.8–1 (large), as adopted in a recent study (Morgado et al., 2021).

Furthermore, we employed a non-parametric Friedman test to examine whether uncertainty modulates (intensifies or dampens) the self-reported intensity of existing emotional states. Follow-up, pairwise Wilcoxon signed rank tests with associated Bonferroni-Holm corrections were conducted to identify significant differences between specific emotions pairs that contributed to the overall significant main effect. Effect sizes were estimated using Kendall’s Coefficient of Concordance “*W”* (Kendall, 1938) for the Friedman test, and the correlation “*r*” for pairwise Wilcoxon tests (Rosenthal, 1991). The effect sizes were interpreted using Cohen’s (1988) guidelines, with both *W* and *r* using the same scale for correlations: 0.1 = small, 0.3 = moderate, and 0.5 = large.

## Results

### Descriptive Statistics

The reported frequencies of the eight emotion categories across the five uncertainty parameters are presented in Table 1. For the descriptive statistics of the self-reported intensity ratings across the six emotional states in relation to uncertainty, please refer to Table 2.

**TABLE 1:** Total frequencies of emotions reported across the five uncertainty parameters.

	General uncertainty	Uncertainty (negative outcomes)	Uncertainty (positive outcomes)	Uncertainty (outcomes can be predicted - risk)	Uncertainty (outcomes cannot be predicted - ambiguity)
					
Emotion	*N*	*N*	*N*	*N*	*N*
Angry/Frustrated	87	106	10	21	96
Confused	137	77	27	29	96
Disgusted	5	14	1	5	10
Excited/Enthusiastic	32	10	164	121	36
Fearful/Anxious	196	202	87	128	197
Happiness/Joyful	15	2	102	60	9
Sadness/Upset	82	114	8	20	71
Surprised/Interested	61	15	121	59	47
Total frequency	**615**	**540**	**520**	**443**	**562**

**TABLE 2:** Descriptive statistics (mean, standard deviation, and median) for self-reported emotion intensity ratings in relation to uncertainty.

Emotion	Mean	SD	Median
Angry/Frustrated	3.72	1.21	4.00
Disgusted	3.06	0.93	3.00
Fearful/Anxious	3.82	1.37	4.00
Excited/Enthusiastic	2.68	1.14	3.00
Happiness/Joyful	2.23	1.01	2.00
Sadness/Upset	3.53	1.27	4.00

### Emotions Associated With General Uncertainty

**FIGURE 1:**
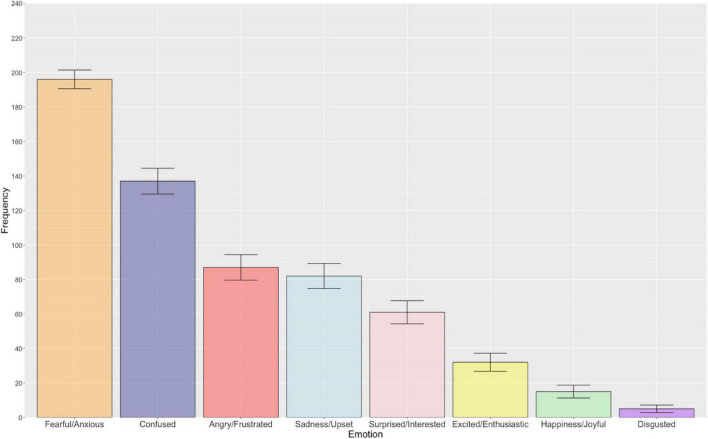
The bar chart displays the frequency of each emotion associated with general uncertainty (descending order). Error bars represent ± 1 standard error.

Figure 1 illustrates the frequency of each emotion category in relation to general uncertainty. Overall, there was a statistically significant difference in the reported frequency with which emotions were associated with general uncertainty [χ^2^(7) = 573.45, *p* < 0.001, *R* = 0.31]. As anticipated, follow-up pairwise McNemar tests revealed that fearful/anxious was significantly more frequently selected than the remaining seven emotions following Bonferroni-Holm correction for multiple comparisons (*p* < 0.001). Broadly, more negative relative to positive emotions were frequently associated with general uncertainty, including the confused and angry/frustrated emotion categories, which exhibited a statistically significant difference from one another (*p* < 0.001, Bonferroni-Holm corrected). All other emotions were found to be significantly different from one another, with the exception of the following emotion pairs that did not survive Bonferroni-Holm correction: angry/frustrated – sadness/upset (*p* = 0.609), sadness/upset – surprised/interested (*p* = 0.124), disgusted – happiness/joyful (*p* = 0.124), and angry/frustrated – surprised/interested (*p* = 0.057). Pairwise comparisons with associated uncorrected and Bonferroni-Holm adjusted *p*-values are presented in the Supplementary Table 3.

### Emotions Associated With Uncertainty in Relation to Negative Outcomes

**FIGURE 2:**
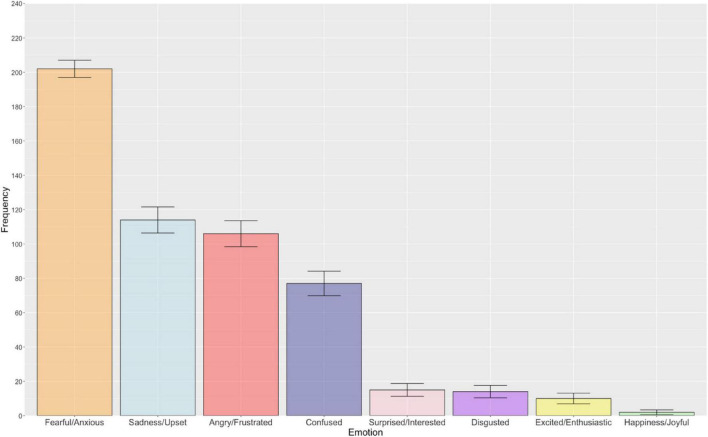
The bar chart displays the frequency of each emotion associated with uncertainty in relation to negative outcomes (descending order). Error bars represent ± 1 standard error.

Figure 2 illustrates the frequency of each emotion category associated with uncertainty in relation to negative outcomes. There was an overall significant difference in the reported frequency of emotions associated with uncertainty in relation to negative outcomes [χ^2^(7) = 713.02, *p* < 0.001, *R* = 0.39]. As hypothesised, *post hoc*, pairwise McNemar tests identified fearful/anxious to be the most frequently reported emotion, which was also significantly different from the seven remaining emotion categories following Bonferroni-Holm correction for multiple comparisons (*p* < 0.001). Other negative emotions that were frequently reported for this particular parameter of uncertainty were sadness/upset and angry/frustrated, which did not demonstrate a statistically significant difference from one another (*p* > 0.999, Bonferroni-Holm corrected). All other emotion categories were significantly different from one another, with the exception of the following emotion pairs that did not survive Bonferroni-Holm correction: disgusted – excited/enthusiastic (*p* > 0.999), disgusted – surprised/interested (*p* > 0.999), excited/enthusiastic – surprised/interested (*p* > 0.999), and excited/enthusiastic – happiness/joyful (*p* = 0.107). Pairwise comparisons with associated uncorrected and Bonferroni-Holm adjusted *p*-values are presented in the Supplementary Table 4.

### Emotions Associated With Uncertainty in Relation to Positive Outcomes

**FIGURE 3:**
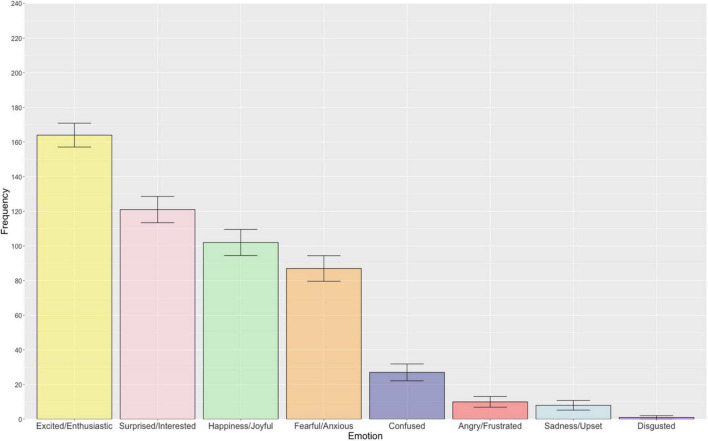
The bar chart displays the frequency of each emotion associated with uncertainty in relation to positive outcomes (descending order). Error bars represent ± 1 standard error.

Figure 3 illustrates the frequency of each emotion associated with uncertainty in relation to positive outcomes. There was an overall significant difference in the reported frequency of emotions associated with uncertainty in relation to positive outcomes [χ^2^(7) = 542.15, *p* < 0.001, *R* = 0.31]. *Post hoc*, pairwise McNemar tests revealed excited/enthusiastic to be significantly more frequently selected relative to the remaining seven emotion categories after Bonferroni-Holm correction for multiple comparisons (*p* < 0.001). Partially supporting our hypothesis, uncertainty in relation to positive outcomes primarily elicited the other positive emotion categories, surprised/interested and happiness/joyful, which were not significantly different from one another (*p* = 0.211, Bonferroni-Holm correction). All other emotions demonstrated a statistically significant difference from one another, with the exception of the following emotion pairs that did not survive Bonferroni-Holm correction: angry/frustrated – sadness/upset (*p* = 0.791), fearful/anxious – happiness/joyful (*p* = 0.503), disgusted – sadness/upset (*p* = 0.156), and disgusted – angry/frustrated (*p* = 0.059). Pairwise comparisons with associated uncorrected and Bonferroni-Holm adjusted *p*-values are presented in the Supplementary Table 5.

### Emotions Associated With the Uncertainty Sub Parameter of Risk

**FIGURE 4:**
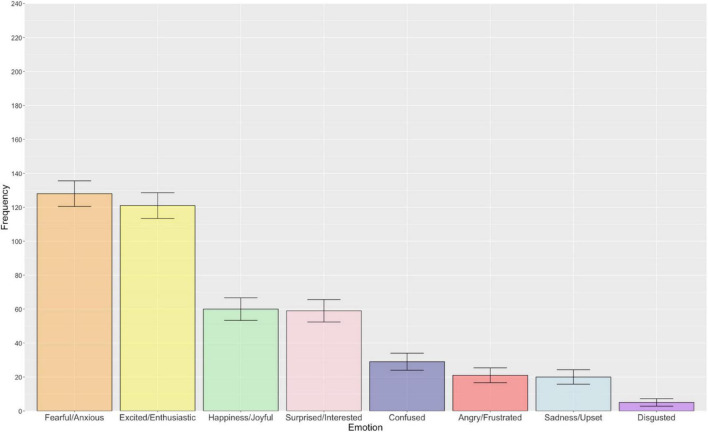
The bar chart displays the frequency of each emotion associated with the uncertainty sub parameter of risk (descending order). Error bars represent ± 1 standard error.

Figure 4 illustrates the frequency of each emotion associated with the uncertainty sub parameter of risk. Overall, there was a statistically significant difference in the reported frequency of emotions in relation to the uncertainty sub parameter of risk [χ^2^(7) = 363.70, *p* < 0.001, *R* = 0.19]. Interestingly, both fearful/anxious and excited/enthusiastic were the most frequently reported emotions and there was no significant difference in their reported frequency following Bonferroni-Holm correction for multiple comparisons (*p* > 0.999). All other emotions demonstrated significant differences from one another, with the exception of the following emotion pairs that did not survive Bonferroni-Holm correction: angry/frustrated – confused (*p* > 0.999), angry/frustrated – sadness/upset (*p* > 0.999), confused – sadness/upset (*p* > 0.999), and happiness/joyful – surprised/interested (*p* > 0.999). Pairwise comparisons with associated uncorrected and Bonferroni-Holm adjusted *p*-values are presented in the Supplementary Table 6.

### Emotions Associated With the Uncertainty Sub Parameter of Ambiguity

**FIGURE 5:**
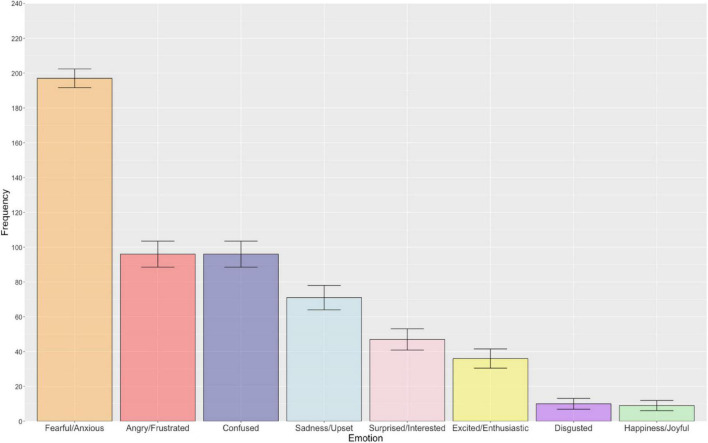
The bar chart displays the frequency of each emotion associated with the uncertainty sub parameter of ambiguity (descending order). Error bars represent ± 1 standard error.

Figure 5 illustrates the frequency of each emotion associated with the uncertainty sub parameter of ambiguity. There was an overall significant difference in the reported frequency of emotions with respect to the uncertainty sub parameter of ambiguity [χ^2^(7) = 544.93, *p* < 0.001, *R* = 0.29]. Fearful/anxious was the most frequently reported emotion and demonstrated statistically significant differences from the other emotion categories following Bonferroni-Holm correction for multiple comparisons (*p* < 0.001). Angry/frustrated and confused were also frequently reported negative emotion categories, which were not found to be significantly different from one another (*p* > 0.999, Bonferroni-Holm corrected). All other emotion types exhibited statistically significant differences from one another, with the exception of the following emotion pairs that did not survive Bonferroni-Holm correction: disgusted – happiness/joyful (*p* > 0.999), excited/enthusiastic – surprised/interested (*p* = 0.509), and sadness/upset – surprised/interested (*p* = 0.081). Pairwise comparisons with associated uncorrected and Bonferroni-Holm adjusted *p*-values are presented in the Supplementary Table 7.

### Uncertainty as a Modulator of Existing Emotional States

**FIGURE 6:**
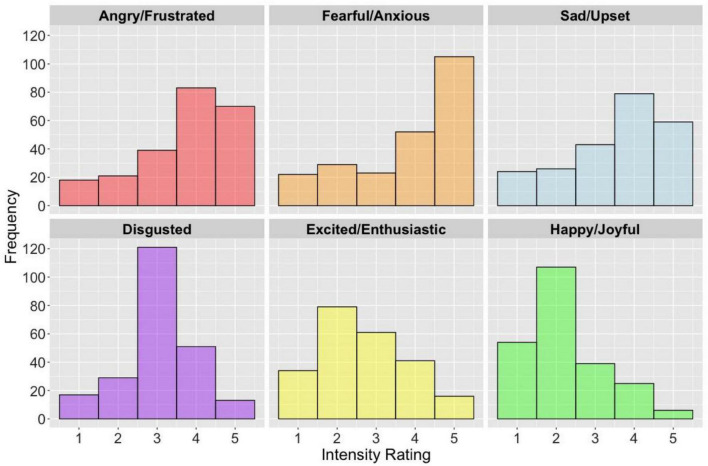
The histograms illustrate the frequency distributions related to the anticipated impact of encountering uncertainty on the self-reported intensity of six discrete emotional states. The intensity ratings range from 1 (uncertainty would make the intensity of this existing emotional state *weaker*) to 5 (uncertainty would make the intensity of this existing emotional state *stronger*). Frequency refers to the number of participants.

Figure 6 displays the frequency distributions of responses related to the anticipated impact of uncertainty on the self-reported intensity of six emotional states. A non-parametric Friedman test was employed to examine the modulatory influence of uncertainty on the self-reported intensity ratings of six emotional states. Overall, there was a statistically significant difference in the self-reported intensity ratings between the six emotional states [χ^2^(5) = 277.44, *p* < 0.001, *W* = 0.24]. *Post hoc* Wilcoxon signed rank tests revealed that upon imagining encountering uncertainty in daily life, fearful/anxious (*Mdn* = 4.0), angry/frustrated (*Mdn* = 4.0) and sadness/upset (*Mdn* = 4.0) exhibited the strongest intensity ratings, suggesting that uncertainty generally increased the self-reported intensity of negative emotional states. Following Bonferroni-Holm correction for multiple comparisons, both fearful/anxious and sadness/upset exhibited a statistically significant difference in intensity rating (*Z* = −4.69, *p* < 0.001, *r* = −0.22), however, there was no significant difference in the intensity ratings between angry/frustrated and fearful/anxious (*Z* = −1.90, *p* = 0.058, *r* = −0.09). The emotion with the lowest rating, indicating that uncertainty weakened the intensity of the emotion, was happiness/joyful, which exhibited statistically significant differences from all other emotions (*p* < 0.001, Bonferroni-Holm corrected). All other emotion pairs exhibited statistically significant differences from one another. Pairwise comparisons with associated uncorrected *p*-values, adjusted Bonferroni-Holm corrected *p*-values, and effect sizes are presented in the Supplementary Table 8.

## Discussion

The current study examined whether uncertainty evokes and modulates a wide range of negative and positive emotions. Uncertainty was predominantly reported to evoke fear/anxiety. However, uncertainty was also reported to evoke a variety of other negative (i.e., sadness/upset, anger/frustration, and confusion) and positive (i.e., surprise/interest and excited/enthusiastic) emotional states depending on the valence of an anticipated outcome (i.e., negative and positive) and the sub parameter of uncertainty (i.e., risk and ambiguity). Furthermore, uncertainty was found to modulate the self-reported intensity of negative and positive emotional experiences, typically heightening the intensity of negative, and dampening the intensity of positive, emotional states. These findings highlight the significance of uncertainty in emotional phenomena, beyond fear and anxiety, which has clear relevance and implications for models of uncertainty and emotion more broadly and in psychopathology.

As hypothesised, uncertainty in general and uncertainty when anticipating a negative outcome was found to primarily evoke fear/anxiety, followed by other negative emotional states (i.e., sadness/upset, anger/frustration, and confusion). These findings are in line with prior theoretical models (Gray, 1990; Hirsh et al., 2012; Brosschot et al., 2016; Carleton, 2016) and empirical research from the appraisal literature (Roseman, 1984; Smith and Ellsworth, 1985) suggesting that uncertainty is aversive in and of itself and engages the behavioural inhibition system responsible for stress and associated negative emotional states. Notably, the findings from this study suggest that the aversive reaction to uncertainty is most commonly expressed as anxiety and fear. However, importantly, the findings from this study also provide further evidence that the aversive reaction to uncertainty is not limited to fear/anxiety and may also be expressed through other negative emotions such as anger/frustration and sadness/upset (Roseman, 1984; Smith and Ellsworth, 1985).

In relation to uncertainty when anticipating a potential positive outcome, we hypothesised that both positive and negative emotional states would be elicited to a similar extent. The present study findings partially support this hypothesis. Uncertainty when anticipating a potential positive outcome primarily elicited positive emotional states, including excitement/enthusiasm, surprise/interest and happiness/joy. This finding supports prior research that has reported uncertainty in the context of positive outcomes to increase curiosity and attention, and to generate and maintain positive emotions, particularly when the outcomes clearly have a positive valence (i.e., uncertainty about which of two potential gifts will be won) (Wilson et al., 2005; Kurtz et al., 2007; Bar-Anan et al., 2009; Anderson et al., 2019). Although, uncertainty in the context of positive outcomes elicited more positive emotional states, it was still associated with eliciting fear/anxiety in over a third of participants. This finding is in agreement with previous research suggesting that some individuals find uncertainty aversive even when there is only potential for positive outcomes (i.e., those with high levels of self-reported Intolerance of Uncertainty) (Carleton, 2016; Pepperdine et al., 2018; Tanovic et al., 2018).

Interestingly, as hypothesised, we found the sub parameters of uncertainty to evoke negative and positive emotional states differently. On the one hand, the uncertainty sub parameter of risk (i.e., when outcomes can be predicted) was found to elicit both positive and negative emotions, specifically high arousal states such as fear/anxiety and excitement/enthusiasm equally. On the other hand, the sub parameter of ambiguity (i.e., when outcomes cannot be predicted) was found to elicit predominantly negative emotions, particularly fear/anxiety. Risk compared to ambiguity, may be viewed as less aversive in general because it is associated with “known” probabilities. The extent to which risk and ambiguity elicit negative and positive emotional states likely differs based on context in which it takes place. In this study, the example was based on employment. Further research is required to examine whether risk and ambiguity differentially evoke negative and positive emotions, depending on the valence of the scenario, i.e., winning the lottery, chance of surviving an illness.

With respect to uncertainty serving as a modulator of emotional states, we hypothesised that experiencing uncertainty in daily life would significantly increase the intensity of existing negative emotional states and reduce the intensity of existing positive emotional states. Our findings provide supporting evidence that uncertainty can both increase and decrease the reported intensity of emotional states, dependent on the valence of the experienced emotion. When individuals imagined encountering uncertainty in their daily lives, uncertainty typically heightened the reported intensity of existing negative emotional states, such as fear/anxiety and anger/frustration, whilst simultaneously dampening the reported intensity of existing positive emotional states, particularly happiness/joy. These findings are partially in line with past research, which has shown uncertainty to both heighten negative affect and dampen positive affect (van Dijk and Zeelenberg, 2006; Bar-Anan et al., 2009). The uncertainty-related valence effects observed in the current study may reflect the generality of the uncertainty we asked participants to imagine (i.e., encountering uncertainty in everyday life). Uncertainty may modulate the intensity of negative and positive emotions differently depending on the sub parameter of uncertainty (i.e., risk and ambiguity), the valence of an anticipated outcome, and the relevance of the uncertainty to the context in which an emotion is expressed (Anderson et al., 2019).

While the findings from this study support current theoretical positions that uncertainty is aversive (Gray, 1990; Hirsh et al., 2012; Brosschot et al., 2016; Carleton, 2016; Peters et al., 2017), it also points to a much-needed expansion of our conceptualisation of uncertainty, in order to account for how uncertainty impacts a wider spectrum of negative and positive emotional states (Anderson et al., 2019; Morriss et al., 2019). The development of a working model of uncertainty that encompasses a broader range of negative and positive emotional states will be particularly informative for understanding how uncertainty and emotion interact in psychiatric disorders such as anxiety and depression. For instance, does the tendency to interpret uncertainty as aversive (i.e., individual differences in Intolerance of Uncertainty, a transdiagnostic dimension: Carleton, 2016) increase the likelihood of experiencing symptoms associated with heightened negative affect (i.e., anxiety, frustration, sadness) and reduced positive affect (i.e., anhedonia) in anxiety and depression? If so, then this will have implications for existing (e.g., Cognitive Behavioural Therapy) and/or new transdiagnostic evidence-based treatments that target uncertainty-related biases to reduce symptoms of anxiety and depression (Shihata et al., 2016).

The study had a few shortcomings, which should be addressed in future research. Firstly, in the current study, we used a relatively crude and simple valence space to define discrete emotion categories. More specific emotions (i.e., anger), typically characterised by higher levels of intensity and certainty, were paired with related, yet distinct emotion labels with a lower intensity and higher degree of uncertainty (i.e., frustration) (Smith and Ellsworth, 1985). Further research should aim to use an expanded dimensional space (for example see, Cowen and Keltner (2017)) to capture subtle nuances between related, but distinct emotional states in relation to the sub parameters of uncertainty (i.e., risk and ambiguity) and the valence of anticipated outcomes (i.e., negative and positive). Secondly, the example scenarios provided in the uncertainty and emotion questionnaire were from the work domain (e.g., exam or job success) because these scenarios involve both negative and positive consequences. However, further research is required to assess whether the pattern of results observed for this study would generalise to example scenarios with different negative and positive consequences (e.g., health, relationships, leisure activities, etc.). Thirdly, the sample consisted of mainly female, White, and heterosexual participants, thus it is unclear whether a similar pattern of results would generalise to other samples. Fourthly, in the present study, the terms used to define emotion and uncertainty were provided in the English language, limiting the diversity of participants who could take part. Replication of the observed effects is warranted in other languages and populations (see Fontaine et al., 2007), in order to improve our understanding of the relevance of this work and its generalisability.

## Conclusion

General uncertainty was predominantly associated with negative emotional states such as fear/anxiety. However, uncertainty was also associated with a variety of other negative (i.e., sadness/upset, anger/frustration, and confusion) and positive (i.e., surprise/interest and excited/enthusiastic) emotional states, depending on the valence of the outcome (i.e., negative and positive) and the sub parameter of uncertainty (i.e., risk and ambiguity). Moreover, uncertainty typically increased the intensity of negative emotional states and decreased the intensity of positive emotional states. These findings highlight that uncertainty is involved in eliciting and modulating a wide array of emotional phenomena, which is informative for the development of working models of uncertainty and emotion more broadly and in relation to psychopathology.

## Data Availability Statement

The datasets presented in this study can be found in online repositories. The names of the repository/repositories and accession number(s) can be found below: All data and analyses have been made publicly available on the Open Science Framework and can be accessed at https://osf.io/yr276.

## Ethics Statement

The studies involving human participants were reviewed and approved by the University of Reading Ethics Committee. The patients/participants provided their written informed consent to participate in this study.

## Author Contributions

JM designed the study and wrote the manuscript. ET conducted the analyses and wrote the manuscript. HD and CH edited the manuscript. All authors contributed to the article and approved the submitted version.

## Author Disclaimer

The views expressed are those of the authors and not necessarily those of King’s College London, NHS, National Institute for Health Research (NIHR), or Department of Health.

## Conflict of Interest

The authors declare that the research was conducted in the absence of any commercial or financial relationships that could be construed as a potential conflict of interest.

## Publisher’s Note

All claims expressed in this article are solely those of the authors and do not necessarily represent those of their affiliated organizations, or those of the publisher, the editors and the reviewers. Any product that may be evaluated in this article, or claim that may be made by its manufacturer, is not guaranteed or endorsed by the publisher.

## Footnotes

1 A *post hoc* power analysis for a one-way, repeated measures, within factors ANOVA based on one group with six measurements (i.e., six emotion categories of interest), with default non-sphericity correction (ε = 1) and correlation among repeated measures (0.5) values, assuming a small to medium effect size (*f* = 0.15) and power of 0.8, indicates that the necessary sample size is 49 participants. Using the aforementioned parameters with eight measurements (i.e., eight emotion categories of interest) shows that the necessary sample size is 41 participants. Power analyses were calculated using G*Power (v3.1).

2
https://www.onlinesurveys.ac.uk/

3 We also ran parametric one-way, repeated measures ANOVAs for the five main parameters of uncertainty. A summary of the main effects and associated effect sizes can be found in the Supplementary Table 2.
