# Supplementary material for: Uncertainty Makes Me Emotional: Uncertainty as an Elicitor and Modulator of Emotional States
Source: Front Psychol. 2022 Mar 8;13:777025. doi: 10.3389/fpsyg.2022.777025 (PMC8957830; doi:10.3389/fpsyg.2022.777025)
Supplement: Supplementary file 1 [file Data_Sheet_1.docx]

**Supplementary Material for “Uncertainty Makes Me Emotional: Uncertainty as an Elicitor and Modulator of Emotional States”**

**Uncertainty and Emotion Questionnaire**

Please identify the emotions that you find relevant to each question by clicking in each box (you can click more than one). If you do not see an emotion that you commonly experience, please type it in the “other: please specify” category. There is no right or wrong answer.

1. Which emotions do you commonly associate with uncertainty generally?

| **Happiness/Joyful** |  |  |
| --- | --- | --- |
| **Sadness/Upset** |  |  |
| **Fearful/Anxious** |  |  |
| **Disgusted** |  |  |
| **Angry/Frustrated** |  |  |
| **Surprised/Interested** |  |  |
| **Excited/Enthusiastic** |  |  |
| **Confused** |  |  |
| **Other** |  |  |
| **If you selected Other, please specify:** |  |  |

1. Which emotions do you commonly associate with uncertainty in relation to potentially negative outcomes (i.e., exam situations, job applications)?

| **Happiness/Joyful** |  |  |
| --- | --- | --- |
| **Sadness/Upset** |  |  |
| **Fearful/Anxious** |  |  |
| **Disgusted** |  |  |
| **Angry/Frustrated** |  |  |
| **Surprised/Interested** |  |  |
| **Excited/Enthusiastic** |  |  |
| **Confused** |  |  |
| **Other** |  |  |
| **If you selected Other, please specify:** |  |  |

1. Which emotions do you commonly associate with uncertainty in relation to potentially positive outcomes (i.e., exam situations, job applications)?

| **Happiness/Joyful** |  |  |
| --- | --- | --- |
| **Sadness/Upset** |  |  |
| **Fearful/Anxious** |  |  |
| **Disgusted** |  |  |
| **Angry/Frustrated** |  |  |
| **Surprised/Interested** |  |  |
| **Excited/Enthusiastic** |  |  |
| **Confused** |  |  |
| **Other** |  |  |
| **If you selected Other, please specify:** |  |  |

1. Which emotions do you commonly associate with uncertainty when you can predict the possible outcomes (i.e., in a job application you know that you will either be successful or unsuccessful)?

| **Happiness/Joyful** |  |  |
| --- | --- | --- |
| **Sadness/Upset** |  |  |
| **Fearful/Anxious** |  |  |
| **Disgusted** |  |  |
| **Angry/Frustrated** |  |  |
| **Surprised/Interested** |  |  |
| **Excited/Enthusiastic** |  |  |
| **Confused** |  |  |
| **Other** |  |  |
| **If you selected Other, please specify:** |  |  |

1. Which emotions do you commonly associate with uncertainty when you can’t predict the possible outcomes because there are many potential outcomes (i.e., your employer is considering merging departments, potentially resulting in change of contract type, new role, promotion, or redundancy)?

| **Happiness/Joyful** |  |  |
| --- | --- | --- |
| **Sadness/Upset** |  |  |
| **Fearful/Anxious** |  |  |
| **Disgusted** |  |  |
| **Angry/Frustrated** |  |  |
| **Surprised/Interested** |  |  |
| **Excited/Enthusiastic** |  |  |
| **Confused** |  |  |
| **Other** |  |  |
| **If you selected Other, please specify:** |  |  |

1. How do you manage uncertainty in your day to day life (can tick more than one answer)?

| **Information Seek** |  |  |  |  |
| --- | --- | --- | --- | --- |
| **Avoid/Withdraw** |  |  |  |  |
| **Accept the uncertainty** |  |  |  |  |
| **Other (please specify)** |  |  |  | |

Please finish each statement by clicking the phrase most characteristic of you. There is no right or wrong answer. Use this scale when answering: (1) Weaker, (2) A Little Weaker, (3) No Difference, (4) A Little Stronger, (5) Stronger.

1. If you were feeling **happy/joyful** would encountering uncertainty in your day to day life make this emotional state

| **Weaker** | **A Little Weaker** | **No difference** | **A Little Stronger** | **Stronger** |
| --- | --- | --- | --- | --- |
| **1** | **2** | **3** | **4** | **5** |

1. If you were feeling **sad/upset** would encountering uncertainty in your day to day life make this emotional state

| **Weaker** | **A Little Weaker** | **No difference** | **A Little Stronger** | **Stronger** |
| --- | --- | --- | --- | --- |
| **1** | **2** | **3** | **4** | **5** |

1. If you were feeling **fearful/anxious** would encountering uncertainty in your day to day life make this emotional state

| **Weaker** | **A Little Weaker** | **No difference** | **A Little Stronger** | **Stronger** |
| --- | --- | --- | --- | --- |
| **1** | **2** | **3** | **4** | **5** |

1. If you were feeling **disgusted** would encountering uncertainty in your day to day life make this emotional state

| **Weaker** | **A Little Weaker** | **No difference** | **A Little Stronger** | **Stronger** |
| --- | --- | --- | --- | --- |
| **1** | **2** | **3** | **4** | **5** |

1. If you were feeling **angry/frustrated** would encountering uncertainty in your day to day life make this emotional state

| **Weaker** | **A Little Weaker** | **No difference** | **A Little Stronger** | **Stronger** |
| --- | --- | --- | --- | --- |
| **1** | **2** | **3** | **4** | **5** |

1. If you were feeling **excited/enthusiastic** would encountering uncertainty in your day to day life make this emotional state

| **Weaker** | **A Little Weaker** | **No difference** | **A Little Stronger** | **Stronger** |
| --- | --- | --- | --- | --- |
| **1** | **2** | **3** | **4** | **5** |

1. In your experience, what situations with uncertainty, if any, have caused you to feel negative emotions?
2. In your experience, what situations with uncertainty, if any, have caused you to feel positive emotions?

| **Table S1.**  Frequency of ‘Other’ Response Across the Five Uncertainty Parameters | |
| --- | --- |
| Uncertainty Parameter | Frequency of “Other” Emotion Response |
| Uncertainty in General | 11 |
| Uncertainty in Relation to Negative Outcomes | 6 |
| Uncertainty in Relation to Positive Outcomes | 7 |
| Uncertainty When Outcomes Can Be Predicted | 22 |
| Uncertainty When Outcomes Cannot Be Predicted | 7 |

| **Table S2.**  Main Effects from the Parametric One-Way Analysis of Variance (ANOVA) Models for the Five Uncertainty Parameters | | | | | |
| --- | --- | --- | --- | --- | --- |
| **Variable** | **Sum of Squares** | ***df*** | **Mean Square** | ***F*** | **η_p_^2^** |
| **Uncertainty in General** |  |  |  |  |  |
| Emotion | 126.39 | 5.18 | 24.41 | 126.39*** | 0.36 |
| Error | 229.99 | 1190.97 | 0.19 |  |  |
| **Uncertainty in Relation to Negative Outcomes** |  |  |  |  |  |
| Emotion | 151.69 | 4.48 | 33.89 | 181.42*** | 0.44 |
| Error | 192.31 | 1029.52 | 0.19 |  |  |
| **Uncertainty in Relation to Positive Outcomes** |  |  |  |  |  |
| Emotion | 115.17 | 4.26 | 27.03 | 116.01*** | 0.34 |
| Error | 228.33 | 980.04 | 0.23 |  |  |
| **Uncertainty When Outcomes Can Be Predicted (Risk)** |  |  |  |  |  |
| Emotion | 66.16 | 5.04 | 13.12 | 66.74*** | 0.23 |
| Error | 227.97 | 1159.42 | 0.20 |  |  |
| **Uncertainty When Outcomes Cannot Be Predicted (Ambiguity)** |  |  |  |  |  |
| Emotion | 114.67 | 4.99 | 23.00 | 116.91*** | 0.34 |
| Error | 225.59 | 1146.49 | 0.20 |  |  |
| *** *p* <.001. Greenhouse-Geisser corrected statistics are reported. | | | | | |

| **Table S3.**  McNemar Pairwise Comparison Tests for Emotions Associated with General Uncertainty | | | |
| --- | --- | --- | --- |
| **Emotion Comparison Pair** | $\boldsymbol{\chi}$**^2^** | ***p*** | ***p_adj*** |
| Angry/Frustrated – Confused | 26.68 | <.001 | <.001 |
| Angry/Frustrated – Disgusted | 78.11 | <.001 | <.001 |
| Angry/Frustrated – Excited/Enthusiastic | 30.70 | <.001 | <.001 |
| Angry/Frustrated – Fearful/Anxious | 94.83 | <.001 | <.001 |
| Angry/Frustrated – Happiness/Joyful | 53.63 | <.001 | <.001 |
| Angry/Frustrated – Sadness/Upset | 0.26 | .609 | .609 |
| Angry/Frustrated – Surprised/Interested | 6.01 | .014 | .057 |
| Confused – Disgusted | 126.18 | <.001 | <.001 |
| Confused – Excited/Enthusiastic | 78.95 | <.001 | <.001 |
| Confused – Fearful/Anxious | 39.58 | <.001 | <.001 |
| Confused – Happiness/Joyful | 103.11 | <.001 | <.001 |
| Confused – Sadness/Upset | 28.31 | <.001 | <.001 |
| Confused – Surprised/Interested | 49.34 | <.001 | <.001 |
| Disgusted – Excited/Enthusiastic | 19.31 | <.001 | <.001 |
| Disgusted – Fearful/Anxious | 189.01 | <.001 | <.001 |
| Disgusted – Happiness/Joyful | - | .041^a^ | .124 |
| Disgusted – Sadness/Upset | 73.11 | <.001 | <.001 |
| Disgusted – Surprised/Interested | 47.27 | <.001 | <.001 |
| Excited/Enthusiastic – Fearful/Anxious | 150.96 | <.001 | <.001 |
| Excited/Enthusiastic – Happiness/Joyful | 7.31 | .007 | .034 |
| Excited/Enthusiastic – Sadness/Upset | 24.50 | <.001 | <.001 |
| Excited/Enthusiastic – Surprised/Interested | 14.79 | <.001 | .001 |
| Fearful/Anxious – Happiness/Joyful | 169.63 | <.001 | <.001 |
| Fearful/Anxious – Sadness/Upset | 99.76 | <.001 | <.001 |
| Fearful/Anxious – Surprised/Interested | 120.51 | <.001 | <.001 |
| Happiness/Joyful – Sadness/Upset | 51.25 | <.001 | <.001 |
| Happiness/Joyful – Surprised/Interested | 28.93 | <.001 | <.001 |
| Sadness/Upset – Surprised/Interested | 3.67 | .055 | .124 |
| ^a^ Binomial distribution used. All other contrasts are continuity corrected. The uncorrected ‘*p’* values reflect the asymptotic *p* value (two-tailed). The ‘*p_adj’* values represent the adjusted, Bonferroni-Holm corrected *p* values. | | | |

| **Table S4.**  McNemar Pairwise Comparison Tests for Emotions Associated with Uncertainty (Negative Outcomes) | | | |
| --- | --- | --- | --- |
| **Emotion Comparison Pair** | $\boldsymbol{\chi}$**^2^** | ***p*** | ***p_adj*** |
| Angry/Frustrated – Confused | 10.45 | .001 | .010 |
| Angry/Frustrated – Disgusted | 82.81 | <.001 | <.001 |
| Angry/Frustrated – Excited/Enthusiastic | 77.80 | <.001 | <.001 |
| Angry/Frustrated – Fearful/Anxious | 70.51 | <.001 | <.001 |
| Angry/Frustrated – Happiness/Joyful | 98.23 | <.001 | <.001 |
| Angry/Frustrated – Sadness/Upset | 0.66 | .416 | >.999 |
| Angry/Frustrated – Surprised/Interested | 70.44 | <.001 | <.001 |
| Confused – Disgusted | 48.66 | <.001 | <.001 |
| Confused – Excited/Enthusiastic | 50.07 | <.001 | <.001 |
| Confused – Fearful/Anxious | 106.04 | <.001 | <.001 |
| Confused – Happiness/Joyful | 69.32 | <.001 | <.001 |
| Confused – Sadness/Upset | 13.94 | <.001 | .002 |
| Confused – Surprised/Interested | 46.51 | <.001 | <.001 |
| Disgusted – Excited/Enthusiastic | - | .541^a^ | >.999 |
| Disgusted – Fearful/Anxious | 180.25 | <.001 | <.001 |
| Disgusted – Happiness/Joyful | - | .004^a^ | .025 |
| Disgusted – Sadness/Upset | 90.75 | <.001 | <.001 |
| Disgusted – Surprised/Interested | 0.00 | >.999 | >.999 |
| Excited/Enthusiastic – Fearful/Anxious | 188.05 | <.001 | <.001 |
| Excited/Enthusiastic – Happiness/Joyful | - | .021^a^ | .107 |
| Excited/Enthusiastic – Sadness/Upset | 86.96 | <.001 | <.001 |
| Excited/Enthusiastic – Surprised/Interested | - | .383^a^ | >.999 |
| Fearful/Anxious – Happiness/Joyful | 198.01 | <.001 | <.001 |
| Fearful/Anxious – Sadness/Upset | 66.40 | <.001 | <.001 |
| Fearful/Anxious – Surprised/Interested | 183.05 | <.001 | <.001 |
| Happiness/Joyful – Sadness/Upset | 106.22 | <.001 | <.001 |
| Happiness/Joyful – Surprised/Interested | - | .002^a^ | .016 |
| Sadness/Upset – Surprised/Interested | 79.37 | <.001 | <.001 |
| ^a^ Binomial distribution used. All other contrasts are continuity corrected. The uncorrected ‘*p*’ values reflect the asymptotic *p* value (two-tailed). The *‘p_adj’* values represent the adjusted, Bonferroni-Holm corrected *p* values. | | |  |
| **Table S5.**  McNemar Pairwise Comparison Tests for Emotions Associated with Uncertainty (Positive Outcomes) | | | |
| **Emotion Comparison Pair** | $\boldsymbol{\chi}$**^2^** | ***p*** | ***p_adj*** |
| Angry/Frustrated – Confused | 8.83 | .003 | .021 |
| Angry/Frustrated – Disgusted | - | .012^a^ | .059 |
| Angry/Frustrated – Excited/Enthusiastic | 142.74 | <.001 | <.001 |
| Angry/Frustrated – Fearful/Anxious | 73.11 | <.001 | <.001 |
| Angry/Frustrated – Happiness/Joyful | 76.68 | <.001 | <.001 |
| Angry/Frustrated – Sadness/Upset | - | .791^a^ | .791 |
| Angry/Frustrated – Surprised/Interested | 93.80 | <.001 | <.001 |
| Confused – Disgusted | 22.32 | <.001 | <.001 |
| Confused – Excited/Enthusiastic | 114.88 | <.001 | <.001 |
| Confused – Fearful/Anxious | 42.45 | <.001 | <.001 |
| Confused – Happiness/Joyful | 46.80 | <.001 | <.001 |
| Confused – Sadness/Upset | 12.00 | <.001 | .004 |
| Confused – Surprised/Interested | 75.87 | <.001 | <.001 |
| Disgusted – Excited/Enthusiastic | 159.06 | <.001 | <.001 |
| Disgusted – Fearful/Anxious | 82.10 | <.001 | <.001 |
| Disgusted – Happiness/Joyful | 97.09 | <.001 | <.001 |
| Disgusted – Sadness/Upset | - | .039^a^ | .156 |
| Disgusted – Surprised/Interested | 116.07 | <.001 | <.001 |
| Excited/Enthusiastic – Fearful/Anxious | 40.97 | <.001 | <.001 |
| Excited/Enthusiastic – Happiness/Joyful | 33.83 | <.001 | <.001 |
| Excited/Enthusiastic – Sadness/Upset | 146.49 | <.001 | <.001 |
| Excited/Enthusiastic – Surprised/Interested | 17.82 | <.001 | <.001 |
| Fearful/Anxious – Happiness/Joyful | 1.32 | .251 | .503 |
| Fearful/Anxious – Sadness/Upset | 75.11 | <.001 | <.001 |
| Fearful/Anxious – Surprised/Interested | 8.64 | .003 | .020 |
| Happiness/Joyful – Sadness/Upset | 81.59 | <.001 | <.001 |
| Happiness/Joyful – Surprised/Interested | 3.27 | .070 | .211 |
| Sadness/Upset – Surprised/Interested | 101.98 | <.001 | <.001 |
| ^a^ Binomial distribution used. All other contrasts are continuity corrected. The uncorrected ‘*p*’ values reflect the asymptotic *p* value (two-tailed). The *‘p_adj’* values represent the adjusted, Bonferroni-Holm corrected *p* values. | | | |
| **Table S6.**  McNemar Pairwise Comparison Tests for Emotions Associated with Uncertainty (Can Predict Outcomes – Risk) | | | |
| **Emotion Comparison Pair** | $\boldsymbol{\chi}$**^2^** | ***p*** | ***p_adj*** |
| Angry/Frustrated – Confused | 1.29 | .256 | >.999 |
| Angry/Frustrated – Disgusted | - | <.001^a^ | .003 |
| Angry/Frustrated – Excited/Enthusiastic | 80.34 | <.001 | <.001 |
| Angry/Frustrated – Fearful/Anxious | 91.35 | <.001 | <.001 |
| Angry/Frustrated – Happiness/Joyful | 22.22 | <.001 | <.001 |
| Angry/Frustrated – Sadness/Upset | 0.00 | >.999 | >.999 |
| Angry/Frustrated – Surprised/Interested | 19.56 | <.001 | <.001 |
| Confused – Disgusted | 16.53 | <.001 | <.001 |
| Confused – Excited/Enthusiastic | 64.70 | <.001 | <.001 |
| Confused – Fearful/Anxious | 83.51 | <.001 | <.001 |
| Confused – Happiness/Joyful | 11.69 | <.001 | .004 |
| Confused – Sadness/Upset | 1.56 | .212 | >.999 |
| Confused – Surprised/Interested | 12.74 | <.001 | .003 |
| Disgusted – Excited/Enthusiastic | 112.08 | <.001 | <.001 |
| Disgusted – Fearful/Anxious | 115.38 | <.001 | <.001 |
| Disgusted – Happiness/Joyful | 47.80 | <.001 | <.001 |
| Disgusted – Sadness/Upset | - | <.001^a^ | .004 |
| Disgusted – Surprised/Interested | 45.31 | <.001 | <.001 |
| Excited/Enthusiastic – Fearful/Anxious | 0.30 | .582 | >.999 |
| Excited/Enthusiastic – Happiness/Joyful | 38.71 | <.001 | <.001 |
| Excited/Enthusiastic – Sadness/Upset | 84.03 | <.001 | <.001 |
| Excited/Enthusiastic – Surprised/Interested | 35.10 | <.001 | <.001 |
| Fearful/Anxious – Happiness/Joyful | 31.61 | <.001 | <.001 |
| Fearful/Anxious – Sadness/Upset | 90.87 | <.001 | <.001 |
| Fearful/Anxious – Surprised/Interested | 38.22 | <.001 | <.001 |
| Happiness/Joyful – Sadness/Upset | 24.53 | <.001 | <.001 |
| Happiness/Joyful – Surprised/Interested | 0.00 | >.999 | >.999 |
| Sadness/Upset – Surprised/Interested | 21.55 | <.001 | <.001 |
| ^a^ Binomial distribution used. All other contrasts are continuity corrected. The uncorrected ‘*p*’ values reflect the asymptotic *p* value (two-tailed). The *‘p_adj’* values represent the adjusted, Bonferroni-Holm corrected *p* values. | | | |
| **Table S7.**  McNemar Pairwise Comparison Tests for Emotions Associated with Uncertainty (Cannot Predict Outcomes - Ambiguity) | | | |
| **Emotion Comparison Pair** | $\boldsymbol{\chi}$**^2^** | ***p*** | ***p_adj*** |
| Angry/Frustrated – Confused | 0.00 | >.999 | >.999 |
| Angry/Frustrated – Disgusted | 78.53 | <.001 | <.001 |
| Angry/Frustrated – Excited/Enthusiastic | 30.54 | <.001 | <.001 |
| Angry/Frustrated – Fearful/Anxious | 84.03 | <.001 | <.001 |
| Angry/Frustrated – Happiness/Joyful | 73.23 | <.001 | <.001 |
| Angry/Frustrated – Sadness/Upset | 9.76 | .002 | .011 |
| Angry/Frustrated – Surprised/Interested | 20.04 | <.001 | <.001 |
| Confused – Disgusted | 78.53 | <.001 | <.001 |
| Confused – Excited/Enthusiastic | 33.47 | <.001 | <.001 |
| Confused – Fearful/Anxious | 81.30 | <.001 | <.001 |
| Confused – Happiness/Joyful | 79.53 | <.001 | <.001 |
| Confused – Sadness/Upset | 7.48 | .006 | .031 |
| Confused – Surprised/Interested | 20.76 | <.001 | <.001 |
| Disgusted – Excited/Enthusiastic | 14.88 | <.001 | .001 |
| Disgusted – Fearful/Anxious | 179.25 | <.001 | <.001 |
| Disgusted – Happiness/Joyful | - | >.999^a^ | >.999 |
| Disgusted – Sadness/Upset | 53.73 | <.001 | <.001 |
| Disgusted – Surprised/Interested | 24.45 | <.001 | <.001 |
| Excited/Enthusiastic – Fearful/Anxious | 143.02 | <.001 | <.001 |
| Excited/Enthusiastic – Happiness/Joyful | 20.49 | <.001 | <.001 |
| Excited/Enthusiastic – Sadness/Upset | 12.99 | <.001 | .002 |
| Excited/Enthusiastic – Surprised/Interested | 1.89 | .170 | .509 |
| Fearful/Anxious – Happiness/Joyful | 180.25 | <.001 | <.001 |
| Fearful/Anxious – Sadness/Upset | 120.19 | <.001 | <.001 |
| Fearful/Anxious – Surprised/Interested | 127.59 | <.001 | <.001 |
| Happiness/Joyful – Sadness/Upset | 51.68 | <.001 | <.001 |
| Happiness/Joyful – Surprised/Interested | 26.33 | <.001 | <.001 |
| Sadness/Upset – Surprised/Interested | 5.40 | .020 | .081 |
| ^a^ Binomial distribution used. All other contrasts are continuity corrected. The uncorrected ‘*p*’ values reflect the asymptotic *p* value (two-tailed). The *‘p_adj’* values represent the adjusted, Bonferroni-Holm corrected *p* values. | | | |

| **Table S8.**  Wilcoxon Pairwise Comparison Tests for Uncertainty and Self-Reported Intensity of Existing Emotional States | | | | |
| --- | --- | --- | --- | --- |
| **Emotion Comparison Pair** | $\mathbf{Z}$ | ***p*** | ***p_adj*** | ***r*** |
| Angry/Frustrated – Disgusted | -8.12 | <.001 | <.001 | -0.38 |
| Angry/Frustrated – Excited/Enthusiastic | -7.67 | <.001 | <.001 | -0.36 |
| Angry/Frustrated – Fearful/Anxious | -1.90 | .058 | .058 | -0.09 |
| Angry/Frustrated – Happiness/Joyful | -9.83 | <.001 | <.001 | -0.46 |
| Angry/Frustrated – Sadness/Upset | -2.79 | .005 | .011 | -0.13 |
| Disgusted – Excited/Enthusiastic | -3.99 | <.001 | <.001 | -0.19 |
| Disgusted – Fearful/Anxious | -7.93 | <.001 | <.001 | -0.37 |
| Disgusted – Happiness/Joyful | -7.59 | <.001 | <.001 | -0.35 |
| Disgusted – Sadness/Upset | -5.74 | <.001 | <.001 | -0.27 |
| Excited/Enthusiastic – Fearful/Anxious | -7.95 | <.001 | <.001 | -0.37 |
| Excited/Enthusiastic – Happiness/Joyful | -6.33 | <.001 | <.001 | -0.29 |
| Excited/Enthusiastic – Sadness/Upset | -6.58 | <.001 | <.001 | -0.31 |
| Fearful/Anxious – Happiness/Joyful | -9.81 | <.001 | <.001 | -0.46 |
| Fearful/Anxious – Sadness/Upset | -4.69 | <.001 | <.001 | -0.22 |
| Happiness/Joyful – Sadness/Upset | -9.04 | <.001 | <.001 | -0.42 |
| The uncorrected ‘*p*’ values reflect the asymptotic *p* value (two-tailed). The ‘*p_adj’* values represent the adjusted, Bonferroni-Holm corrected *p* values. Effect size calculated as: ***r* = Z/√N** (Rosenthal, 1991, p. 19) where N = number of observations. | | | | |

**References**

Rosenthal, R. (1991). *Meta-analytic procedures for social research* (2^nd^ ed.) Newbury Park, CA: Sage.
